# Functional evaluation of novel variants of *B4GALNT1* in a patient with hereditary spastic paraplegia and the general population

**DOI:** 10.3389/fnins.2024.1437668

**Published:** 2024-07-31

**Authors:** Kei-ichiro Inamori, Katsuya Nakamura, Fumi Shishido, Jia-Chen Hsu, Masakazu Nagafuku, Takahiro Nitta, Junji Ikeda, Hidekane Yoshimura, Minori Kodaira, Naomi Tsuchida, Naomichi Matsumoto, Satoshi Uemura, Shiho Ohno, Noriyoshi Manabe, Yoshiki Yamaguchi, Akira Togayachi, Kiyoko F. Aoki-Kinoshita, Shoko Nishihara, Jun-ichi Furukawa, Tadashi Kaname, Masahiko Nakamura, Takayoshi Shimohata, Shu Tadaka, Matsuyuki Shirota, Kengo Kinoshita, Yutaka Nakamura, Isao Ohno, Yoshiki Sekijima, Jin-ichi Inokuchi

**Affiliations:** ^1^Division of Glycopathology, Institute of Molecular Biomembrane and Glycobiology, Tohoku Medical and Pharmaceutical University, Sendai, Japan; ^2^Department of Medicine (Neurology and Rheumatology), Shinshu University School of Medicine, Matsumoto, Japan; ^3^Center for Medical Genetics, Shinshu University Hospital, Matsumoto, Japan; ^4^Faculty of Medicine, Center for Medical Education, Tohoku Medical and Pharmaceutical University, Sendai, Japan; ^5^Laboratory of Bioregulatory Clinical Phamacology, Faculty of Pharmacy, Juntendo University, Urayasu, Japan; ^6^Institute for Environmental and Gender Specific Medicine, Juntendo University Graduate School of Medicine, Urayasu, Japan; ^7^Department of Otorhinolaryngology—Head and Neck Surgery, Shinshu University School of Medicine, Matsumoto, Japan; ^8^Department of Human Genetics, Yokohama City University Graduate School of Medicine, Yokohama, Japan; ^9^Department of Rare Disease Genomics, Yokohama City University Hospital, Yokohama, Japan; ^10^Division of Medical Biochemistry, Faculty of Medicine, Tohoku Medical and Pharmaceutical University, Sendai, Japan; ^11^Division of Structural Glycobiology, Institute of Molecular Biomembrane and Glycobiology, Tohoku Medical and Pharmaceutical University, Sendai, Japan; ^12^Glycan and Life System Integration Center (GaLSIC), Soka University, Hachioji, Japan; ^13^Institute for Glyco-core Research (iGCORE), Nagoya University, Nagoya, Japan; ^14^Department of Genome Medicine, National Center for Child Health and Development, Tokyo, Japan; ^15^Department of Neurosurgery, Matsumoto City Hospital, Matsumoto, Japan; ^16^Department of Neurology, Gifu University Graduate School of Medicine, Gifu, Japan; ^17^Tohoku Medical Megabank Organization, Tohoku University, Sendai, Japan; ^18^Forefront Research Center, Graduate School of Science, Osaka University, Toyonaka, Japan

**Keywords:** hereditary spastic paraplegia, gangliosides, *B4GALNT1*, GM2/GD2 synthase, glycosyltransferase, missense variant

## Abstract

Hereditary spastic paraplegia (HSP) is a heterogeneous group of neurological disorders that are characterized by progressive spasticity and weakness in the lower limbs. SPG26 is a complicated form of HSP, which includes not only weakness in the lower limbs, but also cognitive impairment, developmental delay, cerebellar ataxia, dysarthria, and peripheral neuropathy, and is caused by biallelic mutations in the *B4GALNT1* (beta-1,4-*N*-acetylgalactosaminyltransferase 1) gene. The *B4GALNT1* gene encodes ganglioside GM2/GD2 synthase (GM2S), which catalyzes the transfer of *N*-acetylgalactosamine to lactosylceramide, GM3, and GD3 to generate GA2, GM2, and GD2, respectively. The present study attempted to characterize a novel *B4GALNT1* variant (NM_001478.5:c.937G>A p.Asp313Asn) detected in a patient with progressive multi-system neurodegeneration as well as deleterious variants found in the general population in Japan. Peripheral blood T cells from our patient lacked the ability for activation-induced ganglioside expression assessed by cell surface cholera toxin binding. Structural predictions suggested that the amino acid substitution, p.Asp313Asn, impaired binding to the donor substrate UDP-GalNAc. An *in vitro* enzyme assay demonstrated that the variant protein did not exhibit GM2S activity, leading to the diagnosis of HSP26. This is the first case diagnosed with SPG26 in Japan. We then extracted 10 novel missense variants of *B4GALNT1* from the whole-genome reference panel jMorp (8.3KJPN) of the Tohoku medical megabank organization, which were predicted to be deleterious by Polyphen-2 and SIFT programs. We performed a functional evaluation of these variants and demonstrated that many showed perturbed subcellular localization. Five of these variants exhibited no or significantly decreased GM2S activity with less than 10% activity of the wild-type protein, indicating that they are carrier variants for HSP26. These results provide the basis for molecular analyses of *B4GALNT1* variants present in the Japanese population and will help improve the molecular diagnosis of patients suspected of having HSP.

## 1 Introduction

Hereditary spastic paraplegia (HSP) is a progressive neurodegenerative disease that is characterized by axonal degeneration of the corticospinal tract of the spinal cord, leading to weakness and spasticity in the lower limbs (Shribman et al., 2019; Meyyazhagan and Orlacchio, 2022). It is divided into two main groups: uncomplicated HSP and complicated HSP. Uncomplicated HSP, also known as pure HSP, is characterized by gradual weakness in the legs. Complicated HSP is often accompanied by additional conditions, such as peripheral neuropathy, distal amyotrophy, intellectual disability, ataxia, extrapyramidal symptoms, epilepsy, dysarthria, optic neuropathy, dementia, ichthyosis, learning and developmental disturbances, hearing loss, and speech, breathing, or swallowing difficulties. To date, more than 80 genetic loci have been identified (Meyyazhagan and Orlacchio, 2022). Their genetic loci are referred to as SPG and the subtypes of HSP are designated by the ordinal numbers assigned to their discovery. Various subtypes of HSP exhibit distinct onset ages and disease progression, with some sharing similar conditions.

**Figure 1:**
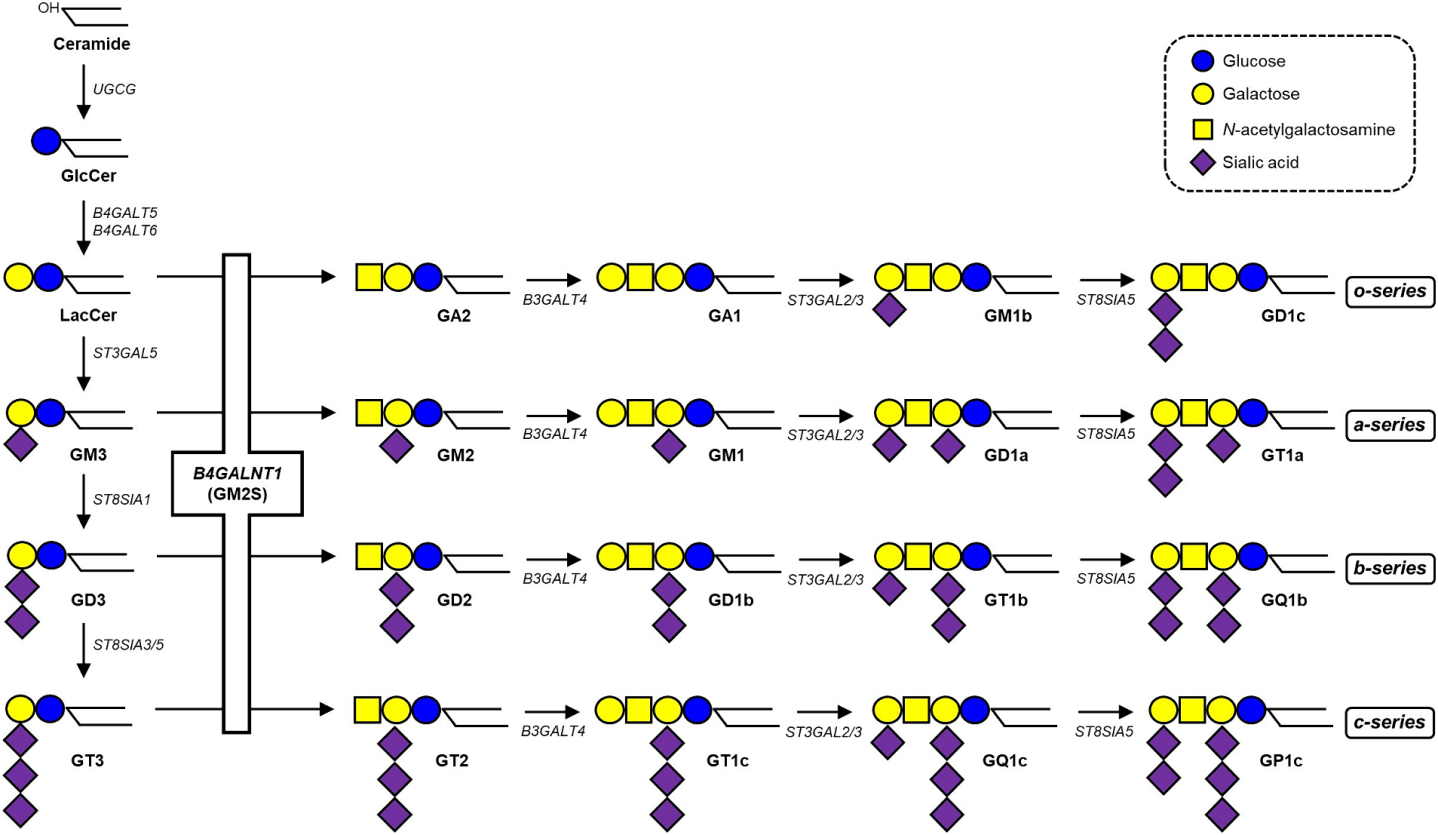
Biosynthetic pathway of gangliosides. See the text for details. The *B4GALNT1* gene encodes GM2/GD2 synthase (GM2S), which catalyzes the transfer of GalNAc to LacCer, GM3, GD3, and GT3 to generate GA2, GM2, GD2, and GT2, respectively.

Glycosphingolipids (GSLs) are integral components of cellular membranes, and sialylated GSLs, which are gangliosides, play crucial roles in various cellular processes (Inokuchi et al., 2018). Ganglioside synthesis is initiated by glucosylceramide synthase (encoded by the *UGCG* gene) to add glucose to ceramide (Ichikawa et al., 1996), followed by the addition of galactose by lactosylceramide (LacCer) synthase (encoded by the *B4GALT5 or B4GALT6* gene; Nomura et al., 1998; Kumagai et al., 2010; Nishie et al., 2010). GM3 synthase (encoded by the *ST3GAL5* gene) adds a sialic acid to initiate the synthesis of a-, b-, and c-series gangliosides (Ishii et al., 1998). GM2/GD2 synthase (GM2S, encoded by the *B4GALNT1* gene) adds *N*-acetylgalactosamine (GalNAc) to LacCer, GM3, GD3, and GT3 to generate GA2, GM2, GD2, and GT2, respectively (Nagata et al., 1992). Other glycosyltransferases synthesize even more complex molecular species downstream (Figure 1). Gangliosides are highly expressed in neural tissues, with the four species, GM1, GD1a, GD1b, and GT1b predominating in the mammalian brain, particularly in the human brain, accounting for 99% of species (Schnaar, 2019). Pathogenic variants in the *ST3GAL5* gene may cause infantile-onset epilepsy syndrome, associated with developmental stagnation and blindness in humans (Simpson et al., 2004). In contrast, mice deficient in the orthologous gene *St3gal5* show no apparent neurological abnormalities, which may be attributed to alternatively expressed o-series gangliosides compensating for the loss of a- and b-series gangliosides (Yamashita et al., 2003; Inamori and Inokuchi, 2022). *B4galnt1* knockout (KO) mice, in which the expression of gangliosides, such as GM3 and GD3, is limited, grow apparently normal; however, myelination decreases as they age and they exhibit progressive axonal degeneration in both the central and peripheral nervous systems (Takamiya et al., 1996; Sheikh et al., 1999; Yao et al., 2014). Pathogenic variants in the human *B4GALNT1* gene cause SPG26, an autosomal recessive (OMIM **#**609195), complicated HSP, characterized not only by lower limb weakness, but also by cognitive impairment, developmental delay, cerebellar ataxia, dysarthria, and peripheral neuropathy, and these clinical phenotypes are similar to those of *B4galnt1* KO mice (Boukhris et al., 2013; Harlalka et al., 2013; Bhuiyan et al., 2019).

In the present study, we performed whole exome sequencing (WES) on our patient with progressive multi-system neurodegeneration and identified a novel, homozygous missense variant in the *B4GALNT1* gene (NM_001478.5:c.937G>A p.Asp313Asn). Functional analyses revealed that the Asp313Asn variant lost its enzyme activity, leading to the diagnosis of SPG26. Furthermore, we analyzed 10 novel missense variants of the *B4GALNT1* gene, which were predicted to be deleterious by the Polyphen-2 and SIFT programs, extracted from the general Japanese population using the whole-genome reference panel of the Tohoku Medical Megabank Organization (ToMMo), for their enzyme activity and subcellular localization.

## 2 Materials and methods

### 2.1 Clinical characterization and molecular testing

Since the clinical findings of our patient were suggestive of slowly progressive multi-system neurodegeneration, we performed WES, after obtaining informed consent. Genomic DNA was extracted from the patient’s peripheral blood. WES was performed as previously described (Inoue et al., 2024). The present study was approved by the Institutional Review Board at Shinshu University. Written consent was obtained. Exome sequencing was conducted at Yokohama City University.

### 2.2 Homology modeling of the B4GALNT1 protein

Homology modeling of the human B4GALNT1 protein was performed using the SWISS-MODEL server (Waterhouse et al., 2018). The crystal structure of the *Drosophila melanogaster* polypeptide *N*-acetylgalactosaminyltransferase PGANT9A in complex with UDP and Mn^2+^ (PDB ID: 6E4Q; Ji et al., 2018) was used as a template. A 3D model of the human structure was displayed using the PyMOL Molecular Graphics System, Version 2.5 Schrödinger, LLC.

### 2.3 Flow cytometry of activated T cells

Peripheral blood mononuclear cells (PBMCs) obtained from the proband, her father (mother’s samples were not obtained), and an age-matched control were stimulated with anti-CD3 and anti-CD28, and the cell surface expression of GM1 on activated T cells was analyzed by flow cytometry using cholera toxin B subunit (CTX), as previously described (Tani-ichi et al., 2005). Briefly, PBMCs were cultured in the presence or absence of anti-CD3 and anti-CD28 for 48 h, and T cells were stained with a combination of antibodies including CD4-FITC, CD8-PE-Cy7, and CD69-APC (BioLegend), and with CTX-CF640R (Biotium).

### 2.4 B4GALNT1-KO HEK293T cells

To generate B4GALNT1-KO cells, human *B4GALNT1* gene-specific guide RNAs were designed using the online CRISPR design tool (Ran et al., 2013). Guide oligonucleotides (5′-CACCGACAAGCCAGAGCGCGTTAG-3′ and 5′-AAACCTAACGCGCTCTGGCTTGTC-3′) were synthesized, annealed, and subsequently inserted into the plasmid pSpCas9(BB)-2A-GFP (Addgene, Cambridge, MA). The resulting plasmid was transfected into HEK293T cells using Lipofectamine 2000 (Thermo Fisher Scientific), according to the manufacturer’s protocol. Clonal cell lines were established through single-cell cloning, and the expression levels of gangliosides were assessed using thin-layer chromatography. In the GM2S enzyme assay, B4GALNT1-KO cells were transiently transfected with the N-terminal FLAG-tagged plasmid, pcDNA3 FLAG-hM2-B4GALNT1. Forty-eight hours after transfection, cells were collected and stored at −80°C until analyzed. In an immunocytochemical analysis, cells were transfected with the pCE puro-hM2-B4GALNT1-FLAG vector containing the C-terminal FLAG-tag, selected with puromycin, and the puromycin-resistant cells obtained were then examined.

### 2.5 GM2S enzyme assay

GM2S enzyme activity was assessed by an HPLC-based assay with a fluorescent-labeled acceptor substrate using microsomal membranes prepared from transfected cells (Inamori et al., 2023). Cells were homogenized in 20 mM 4-(2-hydroxyethyl)-1-piperazineethanesulfonic acid (HEPES) buffer, pH 7.2, and 250 mM sucrose containing 1 mM phenylmethylsulfonylfluoride (PMSF), 1× protease inhibitor cocktail (Nacalai Tesque Inc. Japan). After centrifugation at 6,000 rpm at 4°C for 10 min, the supernatant was ultracentrifuged at 100,000 × *g* at 4°C for 1 h and the resultant pellet (microsomal membranes) was solubilized in 50 mM HEPES buffer, pH 7.2 containing 1× protease inhibitor cocktail, 1 mM PMSF, and 1% Triton X-100. Solubilized microsomal membranes were incubated with 10 μM NBD-GM3 (Peptide Institute, Inc., Osaka, Japan), 1 mM UDP-GalNAc, 10 mM MnCl_2_, 10 mM CDP-choline, and 50 mM HEPES buffer, pH 7.2 at 37°C for 15 min. The reaction was terminated by boiling for 5 min, and the supernatant was analyzed using an LC-18 column (TSKgel ODS-80TM, 5 μm, 4.6 × 250 mm, Tosoh Bioscience, Tokyo, Japan) equipped with an HPLC system (Jasco, Tokyo, Japan). The product was eluted with a gradient of acetonitrile in 50 mM ammonium acetate, pH 4.0. GM2S enzymatic activity was examined in triplicate, calculated as the peak area ratio (product peak area/product peak area + substrate peak area), normalized by the expression level of the B4GALNT1 protein evaluated by Western blotting with the FLAG antibody (DYKDDDDK tag antibody, Fujifilm Wako Pure Chemical Corporation, Osaka, Japan) and anti-calnexin antibody (MBL, Tokyo, Japan), and then calculated with the Volume Tools of ImageLab software (Bio-Rad).

### 2.6 Immunocytochemistry

Transfected cells were cultured on poly-L-lysine-coated coverslips (Sigma-Aldrich), fixed with 3.7% formaldehyde in PBS at room temperature for 15 min, and then rinsed with PBS. Cells were then permeabilized with 0.5% SDS in PBS and treated with Image-iT FX Signal Enhancer (Thermo Fisher Scientific) for 30 min. After that, cells were incubated with a 1:100 dilution of a FLAG antibody (Fujifilm Wako Pure Chemical Corporation) in 1% BSA/PBS for 1 h. Following three washes with PBS, cells were incubated for 30 min with Alexa 488-conjugated anti-mouse IgG antibodies (Thermo Fisher Scientific) diluted to 5 μg/mL in 1% BSA/PBS. Coverslips were then washed three times with PBS, mounted on glass slides using ProLong Gold antifade reagent (Thermo Fisher Scientific), and subjected to a fluorescence microscopy analysis (model spinSR10, Olympus; Tokyo, Japan).

### 2.7 Statistical analysis

Data were analyzed by Dunnett’s multiple comparison test using GraphPad Prism version 7.0.

## 3 Results

### 3.1 Case presentation

**Figure 2:**
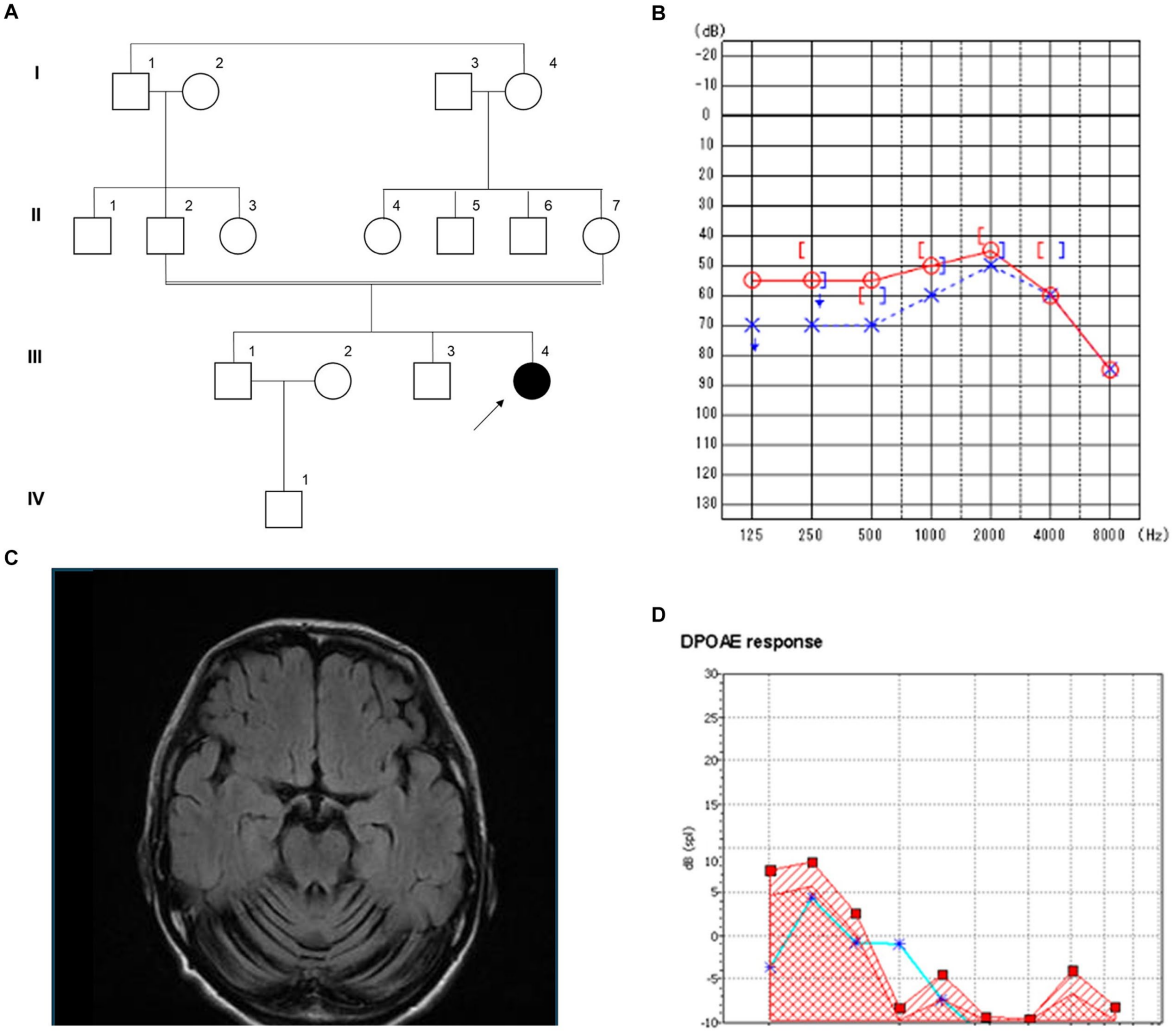
Clinical features of the patient with a novel *B4GALNT1* p.Asp313Asn variant. **(A)** Pedigree of the family. **(B)** Magnetic resonance imaging (MRI) of the brain showed severe cerebellar atrophy. **(C)** Pure tone audiometry. ○: Air conduction, right ear; ×: Air conduction, left ear; [: bone conduction, right ear;]: bone conduction, left ear. **(D)** Results of distortion product otoacoustic emissions of the left ear. From 1 to 8 kHz, with noise denoted by squares and the response denoted by asterisks.

The patient (III-4), a 44-year-old Japanese woman, was the second child of healthy first cousin parents (Figure 2A). Motor and intellectual development was delayed: she held her head up at the age of 8 months, walked without support at 20 months, and spoke words at 24 months. At the age of 3 years, she developed recurrent episodes of unconsciousness with generalized convulsions and, thus, was administered anti-epileptic drugs. At the age of 6 years, mild intellectual disability was noted. At the age of 15 years, she developed paroxysmal episodes of involuntary movement, showing dysesthesia, dysarthria, and the Trousseau sign in both of the upper limbs. At the age of 17 years, extensive neurological examinations were performed for the first time at a local hospital. The findings obtained revealed hypotonia, distal dominant muscle atrophy, and weakness. Tendon reflexes were absent in the upper and lower extremities. Pyramidal tract signs and sensory disturbance were not evident. She walked with a positive waddling gait. Intelligence assessed by the Wechsler Adult Intelligence Scale-Revised test revealed mild intellectual disability with a Full Scale IQ of 60, verbal IQ of 77, and performance IQ scale out. At the age of 19 years, she developed an anxiety disorder and hallucinations and was admitted to a psychiatric hospital. At that time, she was unable to walk without support. At the age of approximately 40 years, she first experienced hearing impairment, which gradually deteriorated. At the age of 42 years, she was referred to our hospital for a more detailed evaluation. Neurological examinations showed normal oculomotor movement. Although speech was slurred, dysphagia was absent. Distal muscle wasting and weakness, with foot deformities were evident. Hypoesthesia and decreases in position and vibration senses were also observed in the limbs. Deep tendon reflexes were absent in the upper and lower extremities. Babinski and Chaddock signs were both positive on both sides. Limb and truncal ataxia were evident. Brain MRI showed marked atrophy of the cerebellar hemispheres and vermis, but not the brainstem. Spinal MRI revealed atrophy of the spinal cord (Figure 2B). The results of nerve conduction studies showed normal motor conduction velocity and compound-muscle action potentials, whereas sensory nerve action potentials were not detected in the upper or lower limbs. The motor-evoked potential in the lower limbs had a prolonged central motor conduction time. Otoscopy revealed no abnormal findings. The results of pure tone audiometry and distortion product otoacoustic emissions indicated mild sensorineural deafness (Figures 2C,D).

### 3.2 Genetic testing and prediction of the impact of the variant

A homozygous missense variant in *B4GALNT1* (NM_001478.5:c.937G>A p.Asp313Asn) was identified. This result was confirmed using Sanger sequencing, and the patient’s non-affected parents were both heterozygous for this variant (Figure 2A). The variant is registered in the Genome Aggregation Database (gnomAD v4.0.0, https://gnomad.broadinstitute.org/) with a minor allele frequency of 0.000007202 (6/833110 alleles). It has not been described in the disease-causing mutation databases, the Human Gene Mutation Database (HGMD) Professional 2024.1^1^ and ClinVar.^2^

**Figure 3:**
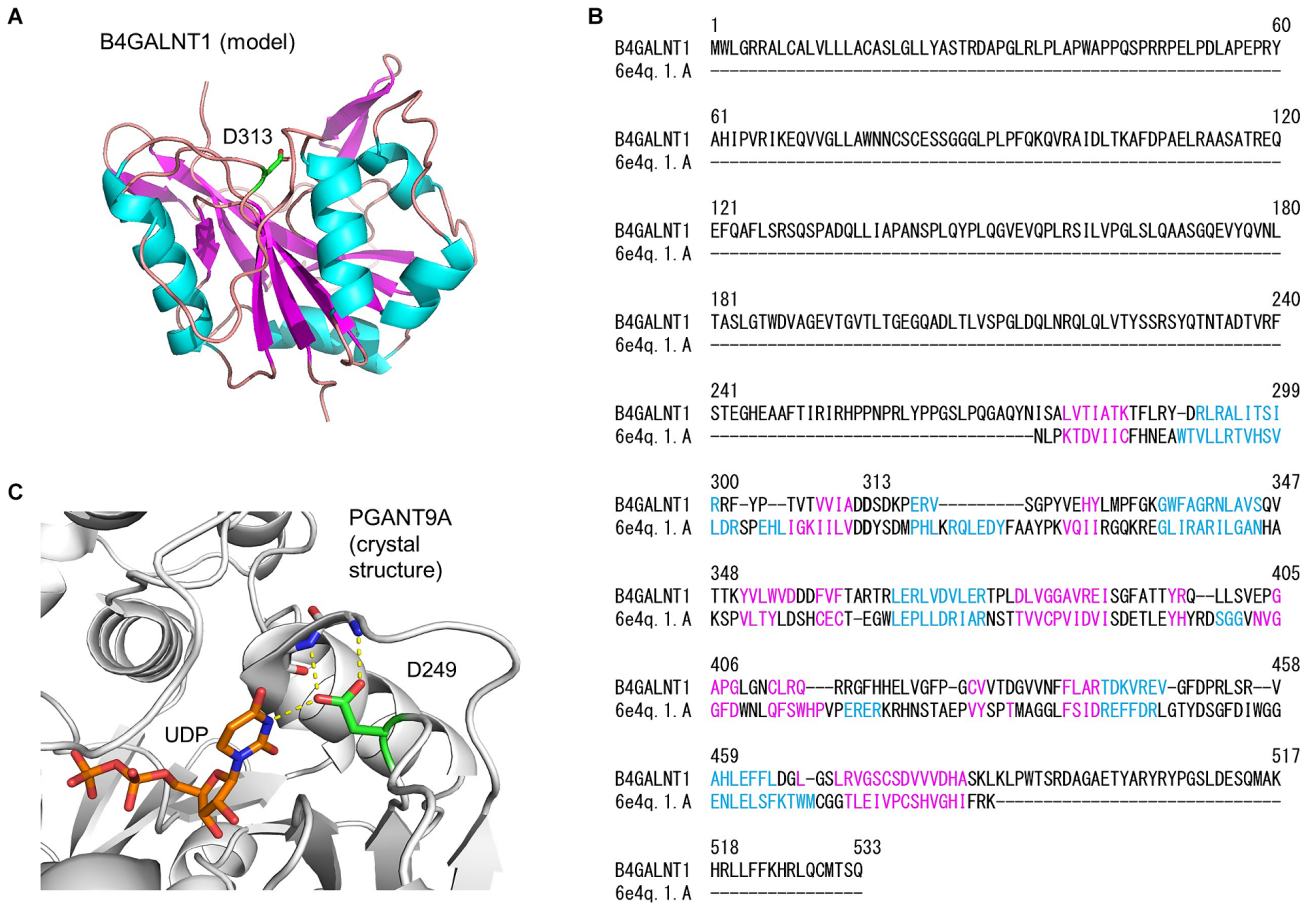
Homology modeling of the B4GALNT1 protein by Swiss Model. **(A)** A 3D model of the human B4GALNT1 catalytic domain. The α-helix (pink) and β-strand (blue) are shown in the ribbon model and Asp313 (D313) is shown in green with the stick representation. **(B)** Alignment of the B4GALNT1 protein sequence and that of the catalytic domain of the template 6E4Q, *Drosophila melanogaster* polypeptide *N*-acetylgalactosaminyltransferase PGANT9A. The sequences shown in pink and blue correspond to the α-helix and β-strand, respectively, in panel **(A)**. **(C)** The crystal structure of the catalytic domain of PGANT9A. The Asp249 (D249) side chain and bound UDP are shown with a stick model and intermolecular hydrogen bonds with dotted yellow lines.

To predict whether the amino acid change, p.Asp313Asn, affected protein function, a theoretical 3D model of the catalytic domain of human B4GALNT1 (GM2S) was constructed with homology modeling using Swiss Model (Figure 3A). *D. melanogaster* polypeptide *N*-acetylgalactosaminyltransferase PGANT9A was used as the template for modeling, and the sequence alignment of B4GALNT1 and the catalytic domain of PGANT9A (Figure 3B) indicated that Asp313 of B4GALNT1 corresponded to Asp249 of PGANT9A, which is a binding site for UDP (Figure 3C). Therefore, Asp313 of B4GALNT1 appeared to be involved in the binding of the donor substrate UDP-GalNAc and the enzyme activity of the p.Asp313Asn variant was predicted to be impaired by the loss of donor binding.

### 3.3 Analysis of cell surface GM1 expression on activated T cells from the patient with the p.Asp313Asn variant

**Figure 4:**
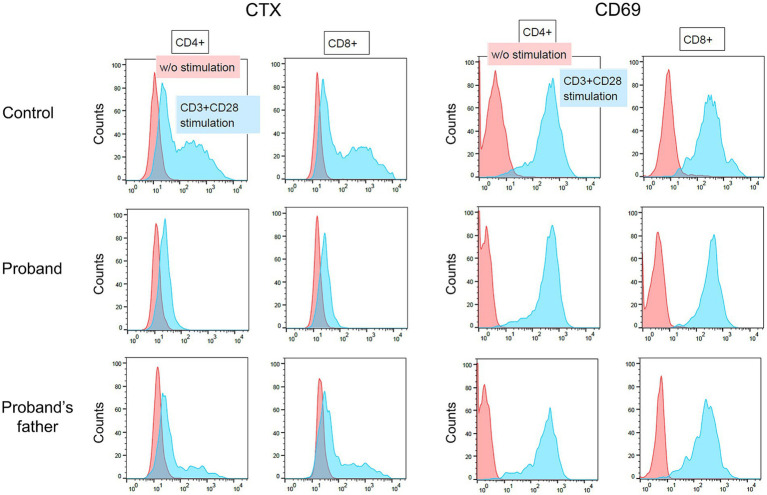
Flow cytometry of activated peripheral blood T cells. Peripheral blood mononuclear cells obtained from an age-matched control male, the patient (proband), and the patient’s father, were cultured with or without anti-CD3 and anti-CD28. Two days later, cells were stained with a combination of antibodies including CD4-FITC, CD8-PE-Cy7, and CD69-APC. CD69 is an activation marker of T cells. The cells surface expression of GM1 was assessed by CTX staining.

In SPG26 patients, pathogenic mutations in the *B4GALNT1* gene result in the loss of GM2S enzyme activity and defective gangliosides biosynthesis (Bhuiyan et al., 2019). We analyzed patient-derived cells to establish whether this defect by the p.Asp313Asn substitution in the B4GALNT1 protein was present. Although human naive T cells express only low levels of GM1 on the cell surface, activation by anti-CD3 and anti-CD28 markedly up-regulates the expression of B4GALNT1, which increases surface GM1 expression (Tani-ichi et al., 2005). We used activation-induced ganglioside expression on T cells to analyze the cell surface expression of GM1 using CTX, which specifically binds to GM1. PBMCs from the patient, her father who is heterozygous for the p.Asp313Asn variant, and a healthy age-matched control male, were stimulated with anti-CD3 and anti-CD28 for 2 days. Surface staining for CD69, an activation marker of lymphocytes, confirmed that T cells were properly stimulated in the experiment (Figure 4). However, GM1 expression was completely impaired in patient T cells, indicating that the p.Asp313Asn substitution resulted in defective ganglioside synthesis.

### 3.4 Functional analysis of the p.Asp313Asn variant protein

**Figure 5:**
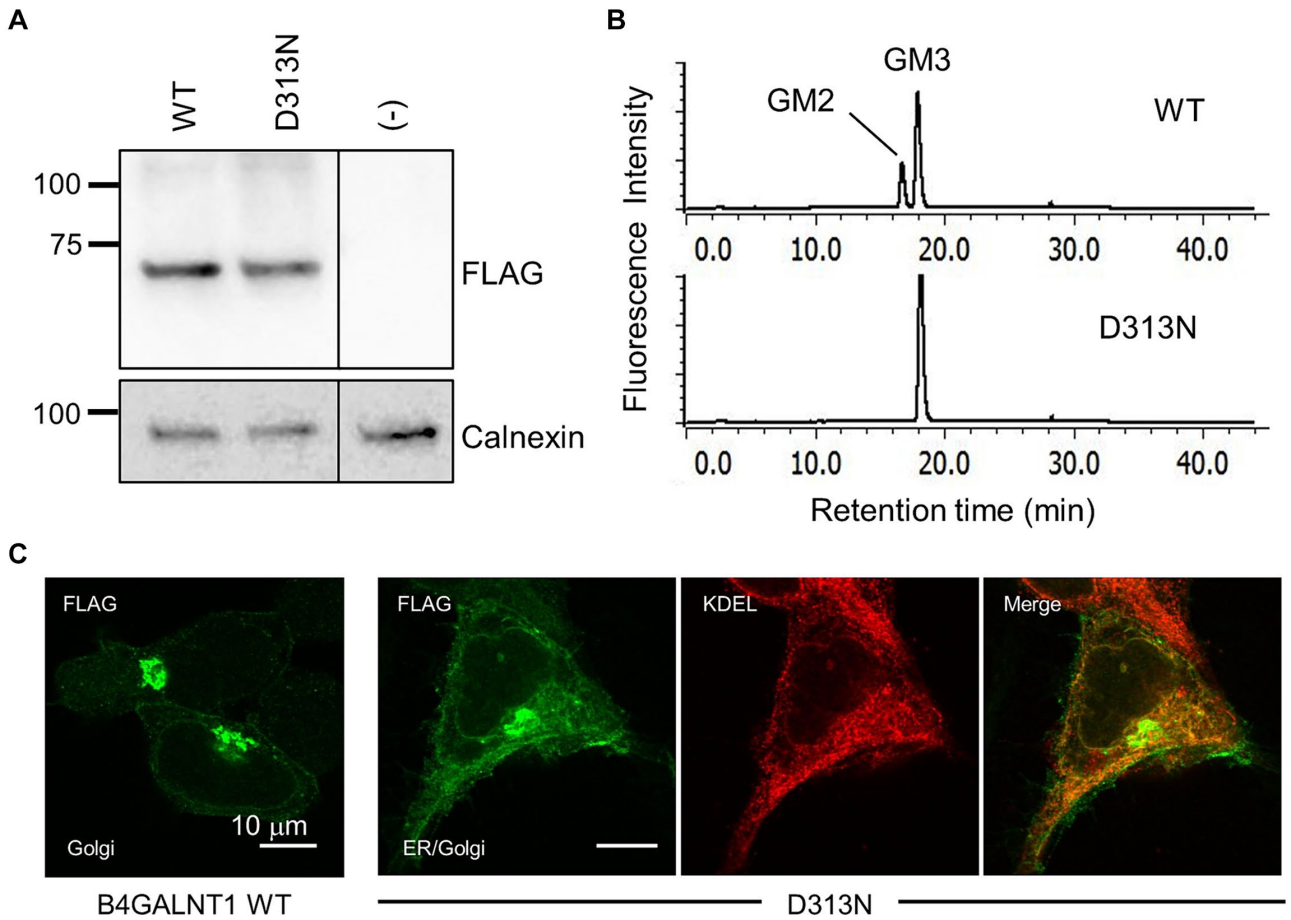
Enzyme activity assay and subcellular localization of the p.Asp313Asn mutant. **(A)** Western blotting for B4GALNT1 proteins transiently expressed in HEK293T/B4GALNT1-KO cells. Anti-FLAG blotting of microsomal membranes collected from cells transfected with B4GALNT1 WT and the p.Asp313Asn (D313N) mutant (upper). The same membrane was blotted with anti-calnexin antibody after stripping to assess the loading amount of membrane proteins (lower). The D313N protein was expressed at a similar level to the WT. **(B)** HPLC chromatograms of the GM2S reaction product. A reaction mixture using fluorescence-labeled GM3 as the substrate was analyzed by HPLC. The reaction was performed with solubilized microsomal membranes from B4GALNT1-KO cells transiently transfected with WT or D313N B4GALNT1. A product peak was not detected in the reaction with D313N. **(C)** Immunohistochemistry for the B4GALNT1 protein in B4GALNT1-KO cells stably expressing WT or D313N. The ER was visualized by staining with the anti-KDEL antibody.

We conducted a functional evaluation of the p.Asp313Asn variant of the B4GALNT1 protein. The N-terminal FLAG-tagged WT or p.Asp313Asn protein was transiently expressed in B4GALNT1-KO HEK293T cells, and GM2S activity was assayed using fluorescent-labeled GM3 (NBD-GM3) as the acceptor substrate and solubilized microsomal membranes as the enzyme source. The WT of B4GALNT1 exhibited GalNAc transferase activity toward GM3, as observed by the product peak (GM2) from the enzyme reaction being eluted earlier than the acceptor substrate peak (GM3; Figure 5A). In contrast, the reaction with p.Asp313Asn showed no product peak, even though the protein was expressed at a similar level to WT, as detected by the FLAG antibody on Western blotting (Figure 5B), demonstrating that the p.Asp313Asn mutant did not exhibit GM2S activity in a detectable range. Similar to numerous other glycosyltransferases, the B4GALNT1 protein localizes to the Golgi apparatus. We examined the subcellular localization of the p.Asp313Asn variant protein stably expressed in B4GALNT1-KO cells. Since the signal sequence required for the endoplasmic reticulum (ER) export and Golgi retention of B4GALNT1 exists at the N terminus (Uemura et al., 2015), a C-terminal FLAG-tagged version of the construct was used for a subcellular localization analysis. Immunocytochemistry for B4GALNT1 stably expressed in KO cells indicated that the p.Asp313Asn protein localized not only to the Golgi, but also to the ER (Figure 5C). This result indicates that Golgi localization was affected in the mutant protein.

### 3.5 Missense variants in the *B4GALNT1* gene extracted from jMorp

To evaluate the GM2S activity of B4GALNT1 variant proteins in the Japanese population, we searched for novel missense variants using the Japanese Multi Omics Reference Panel known as jMorp, a database that contains metabolome and proteome data for plasma obtained from healthy Japanese volunteers from ToMMo (Tadaka et al., 2018). Among the numerous missense variants in the *B4GALNT1* gene, we extracted 11 variants predicted to cause deleterious amino acid substitutions using SIFT and Polyphen2 prediction tools (Adzhubei et al., 2010; Vaser et al., 2016), as shown in Table 1. Ten of the missense variants have not been reported, while one variant, p.D433A, was previously detected in SPG26 patients (Boukhris et al., 2013). Based on the American College of Medical Genetics and Genomics (ACMG) guidelines (Richards et al., 2015), the p.D433A variant was classified as pathogenic, and the other variants were classified as uncertain significance.

**Table 1:** *B4GALNT1* missense variants extracted from jMorp 8.3KJPN.

						Allele frequency	
Position (GRCh37/hg19)	rsID	Transcript consequence	B4GALNT1 variants	PolyPhen2	SIFT	ToMMo 8.3KJPN	gnomAD v2.1.1 Total
12:58025836	rs1408032467	c.80C>A	T27N	D	T	0.0001	NF
12:58025094	rs775744221	c.272T>C	L91P	D	D-LC	0.0001	0.000003978
12:58023984	N/A	c.663A>C	Q221H	D	D-LC	0.0001	NF
12:58022596	rs778379778	c.902G>A	R301H	D	D	0.0002	0.000007953
12:58021487	rs879255242	c.1298A>C	D433A	D	D	0.0001	NF
12:58021474	N/A	c.1311C>G	N437K	D	D	0.0001	NF
12:58021471	N/A	c.1314C>A	F438L	D	D	0.0001	NF
12:58021463	rs563575730	c.1322C>A	A441E	D	D	0.0002	NF
12:58021427	N/A	c.1358C>A	P453H	D	D	0.0001	NF
12:58020574	rs755171392	c.1555C>T	R519W	D	D	0.0001	0.000003976
12:58020553	rs1008314263	c.1576C>T	R526W	D	T	0.0001	0.000003976

D, damaging; T, tolerated; LC, low confidence; NF, not found.

### 3.6 Evaluation of the subcellular localization and GM2S activity of missense variants

**Figure 6:**
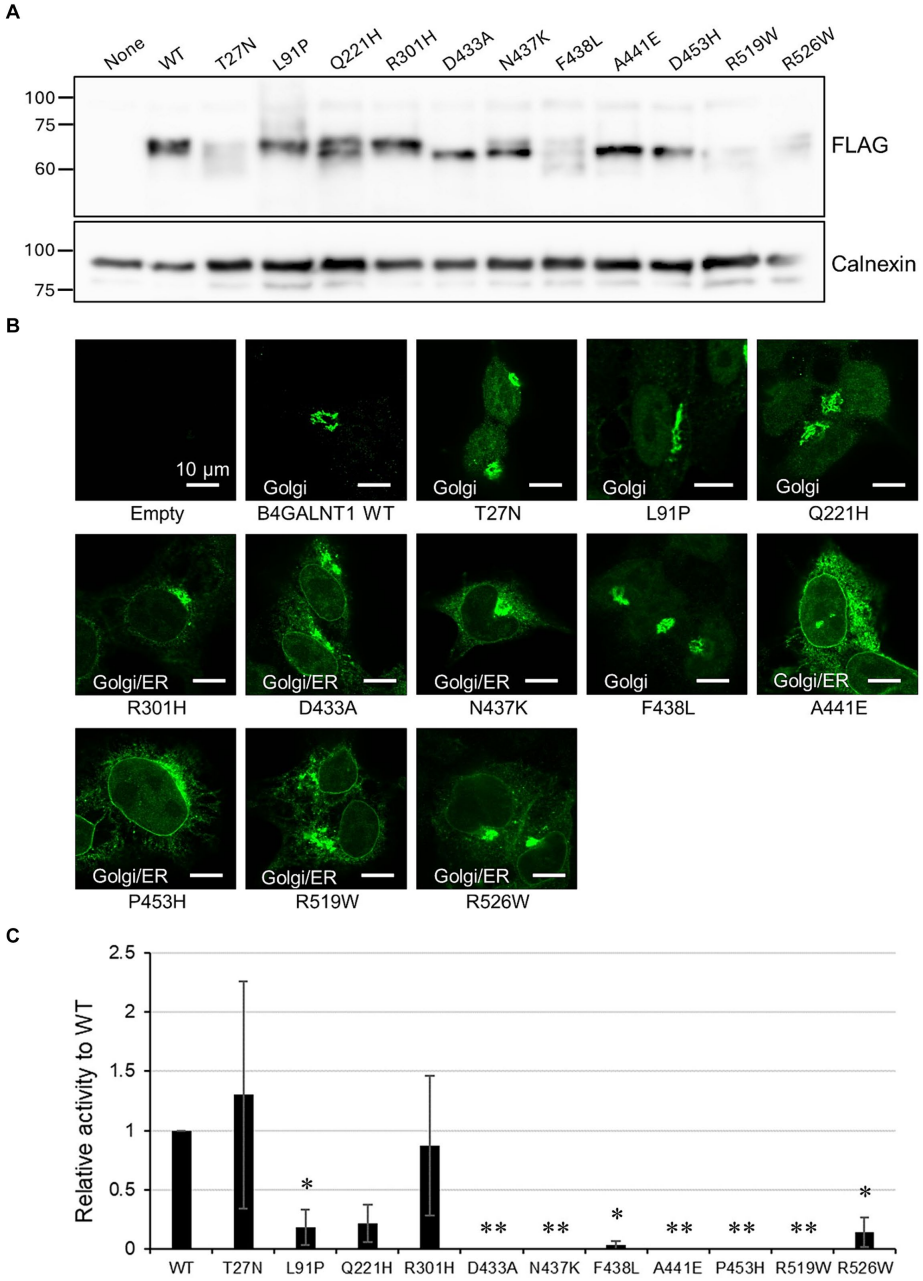
Subcellular localization and GM2S activity of B4GALNT1 variants. **(A)** Western blotting for B4GALNT1 proteins stably expressed in HEK293T/B4GALNT1-KO cells. Calnexin served as the loading control. **(B)** Immunohistochemistry for the B4GALNT1 protein in B4GALNT1-KO cells stably expressing the WT or each variant. **(C)** GM2S assay of the B4GALNT1 WT and variants. The assay was performed as three independent sets of experiments with triplicates in each set, and relative activity with respect to the WT, calculated as the peak area ratio as described in Materials and methods, and the standard deviation in the three experiments are shown. ^*^p < 0.05 vs. WT; ^**^p < 0.01 vs. WT, by Dunnett’s test.

To evaluate the functional impact of missense variants of B4GALNT1 extracted from jMorp, we examined the subcellular localization of the C-terminal FLAG-tagged variant proteins stably expressed in B4GALNT1-KO cells. B4GALNT1-expressing cells obtained by puromycin selection were initially analyzed by Western blotting for protein expression (Figure 6A). Expression levels varied in the variants, suggesting that some of the variants affected protein stability in cells. Interestingly, indirect immunostaining with the FLAG antibody revealed that many of the variants analyzed had both Golgi and ER staining patterns to different degrees (Figure 6B), similar to the p.Asp313Asn variant.

We then assessed the GM2S activities of these variants using microsomal membranes from transiently transfected HEK293T B4GALNT1-KO cells with N-terminal FLAG-tagged constructs. Enzyme activity was normalized by the expression levels of B4GALNT1 proteins assessed by Western blotting (Figure 6C). In the two sets of experiments, a known patients’ mutation p.Asp433Ala showed the loss of enzyme activity as previously reported (Boukhris et al., 2013; Bhuiyan et al., 2019). The novel missense variants, p.Asn437Lys, p.Ala441Glu, p.Pro453His, and p.Arg519Trp, also showed no activity, while p.Phe438Leu exhibited a very weak activity at less than 10% that of WT activity. Since biallelic pathogenic mutations of B4GALNT1 cause SPG26, these novel variants may be carrier mutations.

## 4 Discussion

HSP is a highly heterogeneous group of genetic neurodegenerative disorders, and its pathogenic mechanisms and clinical features vary according to the gene responsible. Next generation sequencing is cost-effective and widely available for diagnoses, but has limitations, and a molecular diagnosis is required to identify the SPG subtype and offer the best therapeutic option to each HSP patient (Shribman et al., 2019; Meyyazhagan and Orlacchio, 2022). SPG26, a complicated form of HSP, is caused by biallelic *B4GALNT1* variants and the clinical phenotypes are similar to those observed in *B4galnt1* KO mice (Takamiya et al., 1996; Sheikh et al., 1999; Boukhris et al., 2013; Harlalka et al., 2013). The GM2S activity of the B4GALNT1 protein is required for the biosynthesis of the complex gangliosides GD1a and GT1b, which are primarily expressed on axonal membranes and serve as receptors for myelin-associated glycoprotein (MAG; Yang et al., 1996; Collins et al., 1997). MAG is also known as Siglec-4, a member of the Siglec family that comprises sialic acid-binding immunoglobulin-like lectins (Macauley et al., 2014), is expressed on the innermost myelin sheath in oligodendrocytes and Schwann cells, and functions in bidirectional axon–glia signaling and inhibiting axon regeneration to maintain myelinated axons (Quarles, 2007; Lopez, 2014). MAG KO mice exhibited modest nervous system abnormalities, similar to *B4galnt1* KO mice (Li et al., 1994; Montag et al., 1994; Takamiya et al., 1996; Sheikh et al., 1999), while MAG/*B4galnt1* double KO mice had similar neuropathological phenotypes to single KO mice without exacerbation (Pan et al., 2005), supporting the contribution of the MAG-ganglioside interaction to axon-myelin stability. The combination of the *cis* homodimerization of MAG and *trans* interactions with gangliosides were identified as the mechanisms underlying MAG functions (Pronker et al., 2016).

In the present study, we found a novel missense variant (c.937G>A p.Asp313Asn) in the *B4GALNT1* gene from a patient with progressive multi-system neurodegeneration. The clinical features observed in our patient were similar to those in previously reported patients with HSP26 (Boukhris et al., 2013). Although the crystal structure of the B4GALNT1 protein has not yet been reported, a prediction based on homology modeling suggested the involvement of the Asp313 residue in the binding of the donor substrate UDP-GalNAc, which is required for glycosyltransferase activity. A functional analysis, including the GM2S enzymatic assay, using patient-derived cells and the expression of the B4GALNT1 protein in cultured cells revealed that the amino acid substitution (p.Asp313Asn) caused the loss of GM2S enzyme activity, leading to the molecular diagnosis of SPG26. This is the first SPG26 case to be reported in Japan. To expand our understanding of SPG26 in the Japanese population, we extracted missense *B4GALNT1* variants from jMorp (ToMMo 8.3KJPN) that were predicted to be deleterious by the SIFT and PolyPhen2 programs performed a functional analysis of these variant proteins expressed in B4GALNT1-KO cells. In a previous study by Bhuiyan et al. (2019), the GM2S enzyme activity and subcellular localization of mutant B4GALNT1 proteins for 11 genetic mutations previously reported in SPG26 patients were examined. Four were missense variants, including p.Asp433Ala, which was also analyzed in the present study. They demonstrated that the majority of mutations resulted in the loss of enzyme activity, which may be responsible for the clinical features of HSP. One of the missense mutants, p.Arg300Cys, exhibited weak activity (approximately 10% that of the WT protein). In their study, these mutants as well as the WT protein exhibited similar Golgi localization patterns, evaluated by the transient transfection of HEK293T cells with C-terminal myc-His_6_-tagged B4GALNT1 constructs (Bhuiyan et al., 2019). In the present study, among the novel variants extracted from jMorp, p.Asn437Lys, p.Ala441Glu, p.Pro453His and p.Arg519Trp did not exhibit enzyme activity and p.Phe438Leu showed less than 10% of the activity of the WT, indicating that these are pathogenic variants. A missense variant found in a HSP patient that caused the amino acid substitution of Arg519 to Pro has been also reported to result in the loss of enzyme activity (Alecu et al., 2022). The immunocytochemical analysis using stable cells expressing C-terminal FLAG-tagged B4GALNT1 proteins showed that most of the missense variants localized not only to the Golgi, but also the ER, as was the case with the p.Asp313Asn variant, indicating that each single amino acid substitution may affect export from the ER. Changes in protein folding due to the amino acid substitution may trigger the quality control of ER export, which monitors the folding status of proteins captured in COPII vesicles by cargo receptors (Phillips et al., 2020). Therefore, the missense B4GALNT1 variants examined in the present study may partially affect ER export.

Consistent with the previous findings (Tani-ichi et al., 2005) and as demonstrated in the present analysis of the peripheral blood T cells from our HSP patient and controls (Figure 3), activation by anti-CD3/CD28 markedly enhanced *B4GALNT1* gene expression and GM2S activity, resulting in the up-regulated expression of GM1 in T cells. Therefore, the use of activated peripheral blood T cells may contribute to a molecular diagnosis and facilitate carrier screening for deleterious *B4GALNT1* variants. In conclusion, our functional studies on novel *B4GALNT1* variants confirmed a loss-of-function, leading to a diagnosis of SPG26, and the identification of unaffected variant carriers. Our approach will contribute to the clinical diagnosis of patients with HSP.

## Data availability statement

The datasets presented in this article are not readily available because they contain personal data. Requests to access the datasets should be directed to the corresponding author.

## Ethics statement

The studies involving humans were approved by the Ethics Committee of Shinshu University School of Medicine/the Ethics Committee of Tohoku Medical and Pharmaceutical University. The studies were conducted in accordance with the local legislation and institutional requirements. The participants provided their written informed consent to participate in this study. Written informed consent was obtained from the individual(s) for the publication of any potentially identifiable images or data included in this article.

## Author contributions

K-iI: Conceptualization, Data curation, Funding acquisition, Investigation, Supervision, Writing – original draft, Writing – review & editing. KN: Data curation, Funding acquisition, Investigation, Resources, Writing – review & editing. FS: Investigation, Writing – review & editing. J-CH: Investigation, Writing – original draft. MNag: Investigation, Writing – review & editing. TN: Investigation, Writing – review & editing. JI: Investigation, Writing – review & editing. HY: Investigation, Writing – review & editing. MK: Investigation, Writing – review & editing. NT: Data curation, Funding acquisition, Investigation, Writing – review & editing. NaM: Funding acquisition, Supervision, Writing – review & editing. SU: Investigation, Writing – review & editing. SO: Formal analysis, Writing – review & editing. NoM: Formal analysis, Writing – review & editing. YY: Formal analysis, Writing – review & editing. AT: Writing – review & editing. KA-K: Resources, Software, Writing – review & editing. SN: Writing – review & editing. J-iF: Writing – review & editing. TK: Resources, Writing – review & editing. MNak: Resources, Writing – review & editing. TS: Resources, Writing – review & editing. ST: Software, Writing – review & editing. MS: Resources, Writing – review & editing. KK: Resources, Software, Writing – review & editing. YN: Project administration, Writing – review & editing. IO: Project administration, Writing – review & editing. YS: Supervision, Writing – review & editing. J-iI: Funding acquisition, Supervision, Writing – review & editing.

## Footnotes

^1^ http://www.hgmd.org/

^2^ http://www.ncbi.nlm.nih.gov/clinvar/
